# Preparation and Improved Physical Characteristics of Propylene Oxide Rubber Composites

**DOI:** 10.3390/molecules23092150

**Published:** 2018-08-27

**Authors:** Natalia N. Petrova, Viktoriya V. Portnyagina, Vasilii V. Mukhin, Ee Le Shim, Jin-Ho Cho

**Affiliations:** 1 Department of Chemistry, North-Eastern Federal University, Yakutsk 677000, Russia; mvvnj@yandex.ru; 2 Mining Institute, North-Eastern Federal University, Yakutsk 677000, Russia; vick_i@mail.ru; 3 School of Mechanical & Automotive Engineering, Halla University, Wonju 220-712, Korea; elshim@halla.ac.kr; 4 Department of Energy Science and Technology, Myongji University, Yongin 449-728, Korea

**Keywords:** propylene oxide rubber, ultrafine polytetrafluoroethylene, composite materials, frost resistance, wear resistance, hydrocarbon resistance

## Abstract

Sealing rubbers employed in cold climates such as the Siberian Arctic must be able to withstand temperatures as low as −50 °C while still exhibiting specific relaxation, strength, tribological characteristics, and a resistance to aggressive media. Previous investigations of propylene oxide rubber (SKPO, T_g_ = −73 °C) modified with polytetrafluoroethylene (PTFE) have revealed that, while the rubber composite materials exhibit double the wear resistance compared to unmodified polypropylene oxide rubber, they have poor frost resistance. In the present study, we developed materials based on SKPO and ultrafine PTFE (UPTFE), which can be characterized by its smaller particle size, low molecular weight, high tribo-technical characteristics, and resistance to aggressive media. The properties of the rubber composites were evaluated using the standard methods. The structures of the materials were investigated by electron microscopy and XRD analysis. It was shown that the materials have excellent wear resistance, resistance to aggressive media, compression set, and low-temperature resistance. The addition of UPTFE is preferable to the addition of PTFE because the desired positive effects can be attained with only 0.5–1 parts per hundred parts of rubber (phr) UPTFE while 20 phr PTFE would be necessary.

## 1. Introduction

Sealing rubbers must have high strength, high relaxation characteristics, excellent resistance to hydrocarbons, and good tribological characteristics [1,2] given that they are used in control machinery, feed machinery, power machinery, and other machinery systems that are exposed to pressure oscillations, pulse loads, liquid-hydrocarbon environments, and severe climates. In particular, the sealing rubbers employed in the Siberian arctic must be frost-resistant to very low temperatures (<−55 °C) despite rubber materials generally becoming fragile once the ambient temperature drops to their glass transition temperature (T_g_). The T_g_ of most commercial rubbers is above −50 °C. For this reason, ordinary rubbers are not used in artic regions. Currently, butadiene nitrile rubber with a low acrylonitrile content is used because its T_g_ is about −50 °C [3,4]. However, this rubber can still only offer limited resistance to hydrocarbon media and is easily damaged under actual arctic operating conditions because prolonged exposure to extremely low temperatures (down to −55 °C) as well as high annual (100 °C) and daily (25–30 °C) temperature differences in the Siberian region have a significant impact on the reliable operation of machinery [1,2,5]. Faced with such conditions, the operating efficiency of the machinery is highly dependent on the sealing parts. For example, in the Republic of Sakha (Yakutia), up to 30% of machinery failure cases in the fields of road transport, mining equipment, and pipelines are a result of the destruction or elasticity loss of rubber sealing parts [1]. In this respect, research to determine the most efficient modifiers and the development of new rational compositions of rubbers with a high level of frost, oil, and wear resistance is crucial. More specifically, the creation of new elastomeric sealing materials exhibiting high performance under severe Siberian arctic conditions is vital for industries engaged in the development of mineral resources such as oil and gas as well as the related processing activities. 

Propylene oxide rubber (SKPO, specific grade, TU 2294-067-16810126-99), which is a copolymer of propylene oxide and 2.5% allyl glycidyl ether, was commercialized in 2002 by the JSC Sterlitamak Petrochemical Plant. It was developed specifically for cold climates with SKPO having an extremely low T_g_ (−74 °C) [6]. Several investigations in which SKPO was modified with 20 parts per hundred parts of rubber (phr) [7] of PTFE revealed that the rubber composite material exhibits a 15% greater oil resistance and double the wear resistance compared to unmodified polypropylene oxide rubber but exhibits poor frost resistance [8,9,10]. Consequently, an SKPO/PTFE composite was not sufficiently resilient to use in the severe Siberian environment. 

UPTFE (brand name: “Forum”) is a PTFE with a molecular weight that is relatively lower than that of ordinary PTFE [11,12,13,14,15,16,17,18,19,20]. UPTFE has unique thermal and aggressive media resistance, chemical resistance, a low coefficient of friction, and can withstand temperatures of −169 °C to 270 °C [13,14]. UPTFE particles are spherical with a diameter of 0.5 µm to 4.5 µm while the diameter of ordinary PTFE particles is 1.8 µm to 10 µm [12]. UPTFE is less heat resistant and more soluble in polar solvents such as acetone and alcohol than ordinary PTFE. Therefore, UPTFE exhibits enhanced adhesion to metal surfaces. UPTFE is used as an additive in oils because the UPTFE particles become firmly bonded to metal surfaces and fill any surface roughness with a dense film. Thus, this reduces the friction coefficient between the metal surfaces and protecting those surfaces from wear and corrosion [11]. A similar surface action mechanism would most likely occur if UPTFE were to be added to SKPO-based rubber composites because the smaller size of the UPTFE particles in the rubber composites should result in superior properties. In the present study, propylene oxide rubber composites with UPTFE (“Forum” brand, TU 2229-004-02698192-2002) [11,12,13,14] was investigated to achieve oil, wear, and frost resistance as well as enhanced mechanical properties in our ongoing effort to devise a material that can be applied even in arctic conditions.

## 2. Materials and Methods

SKPO-UPTFE (JSC “Sterlitamak Petrochemical Plant”, Sterlitamak, Russia) rubber composites [6] were produced using the same process as that used to produce standard SKPO rubber composites. As shown in Table 1, the SKPO-UPTFE rubber composites contain several ingredients such as a dispersant, sulfur, vulcanization accelerator, activator, filler, and an antioxidant. SKPO-UPTFE rubber composites were blended in a Plasticorder (Brabender PL-2200-3, Duisburg, Germany) at 35 °C to 70 °C for 20 min while maintaining the phr concentration of the other constituents. First, SKPO was placed in the mixer together with the dispersant, zinc oxide, and the curing group. At the 9-min mark, UPTFE was added. Then, carbon black, plasticizer, and sulfur were added at 17 min shortly before the end of blending. After 24 h, the SKPO-UPTFE rubber composite was cured at 150 °C for 30 min in a hydraulic press. 

The main technical characteristics of the resulting SKPO-UPTFE rubber composites were evaluated according to Russian standard methods (GOST 270-84, GOST 9.029-74, GOST 408-78, GOST 9.030-74 [21,22,23,24]). The wear resistance of the SKPO-UPTFE rubber composites was evaluated by the abrasion resistance determination method using an AR-40 device [25]. The structural characteristics of the SKPO-UPTFE rubber composites were investigated by using a scanning electron microscope (JEOL, JSM–6480LV, Tokyo, Japan) equipped with an EDS detector (Oxford, Х-mах20, High Wycombe, UK) as well as an X-ray powder diffractometer (Thermo Fisher Scientific, ARL X’TRA, Écublens, Switzerland,) with copper as the anode. The scan angle ranged from 3° to 60° while the step angle was 0.04°. The degree of crystallinity was determined to be the Thermo-Fisher crystallinity. The frost resistance of the rubber composites was tested according to the GOST 408-78 standard [23] where the frost resistance coefficient is the ratio of the elastic modulus at room temperature at a particular temperature. 

## 3. Results and Discussion

Table 1 lists the compositions of standard SKPO rubber, SKPO-PTFE rubber composite, and SKPO-UPTFE rubber composite.

The physical and mechanical properties of blended SKPO-UPTFE rubber composites were evaluated and are summarized in Table 2. 

UPTFE is a more rigid polymer with a higher modulus than SKPO. Larger amounts of UPTFE lead to a reduction in elasticity due to a decrease in the flexibility and the mobility of the propylene oxide rubber. These factors decrease the elongation at break. A significant amount (20 phr) of PTFE (rigid polymer) in SKPO inhibits the development of high-elastic deformation at both room and low temperatures, which leads to the sharp decrease in the frost-resistance coefficient (K_V_). Because the particle size of UPTFE is much smaller than that of PTFE, the SKPO-UPTFE rubber composite exhibits a frost resistance of >0.90. Small particles of UPTFE with a low coefficient of friction can also be regarded as being a dry lubricant, which facilitates the slipping of the SKPO macromolecules and restructuring under deformation at low temperatures. The T_g_ value of standard SKPO rubber was 64.6 °C while the T_g_ values of all the SKPO-UPTFE rubber composites were no higher than that of the standard SKPO rubber.

In general, SKPO-UPTFE rubber composites exhibit significant improvements in wear and oil resistance, relaxation properties (compression set), and frost resistance at low UPTFE filler dosages (<5 phr). The best SKPO-UPTFE rubber composite was obtained with 1 phr UPTFE to the SKPO, which is shown in Table 2. With this composition, the volumetric wear, degree of swelling, and compression set are reduced by 41%, 22%, and 11%, respectively, while the frost resistance coefficient at −50 °C is increased by 12% relative to that of standard SKPO rubber. As shown in Table 2, the addition of small amounts (0.5–1 phr UPTFE) relative to a large amount (20 phr) of PTFE was enough to improve the physical and mechanical properties [9,10]. The enhanced properties cannot be explained by the low friction coefficient of the added UPTFE alone and require further detailed study.

**Figure 1:**
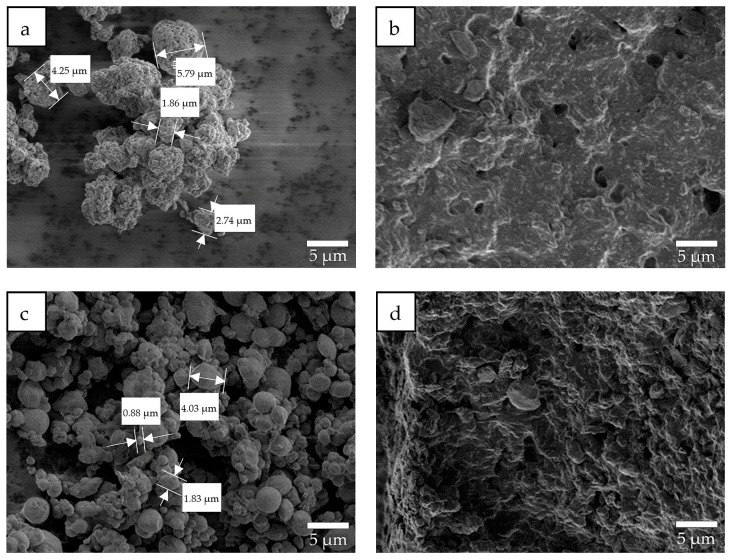
SEM images (× 3000): (**a**) PTFE, (**b**) SKPO + 10 phr PTFE, (**c**) UPTFE, and (**d**) SKPO + 10 phr UPTFE.

SEM images of the standard SKPO rubber, SKPO-PTFE rubber composite, and SKPO-UPTFE rubber composites were investigated to evaluate the structural changes. The external surface as well as the interior of the rubber composites (obtained by dipping them in liquid nitrogen and then crushing them) were investigated. The ordinary PTFE particles were found to be significantly larger than the UPTFE particles, which is shown in Figure 1a,c. Both the PTFE and UPTFE have similar polymeric structures before and after their addition to SKPO rubber, which is shown in Figure 1b,d.

**Figure 2:**
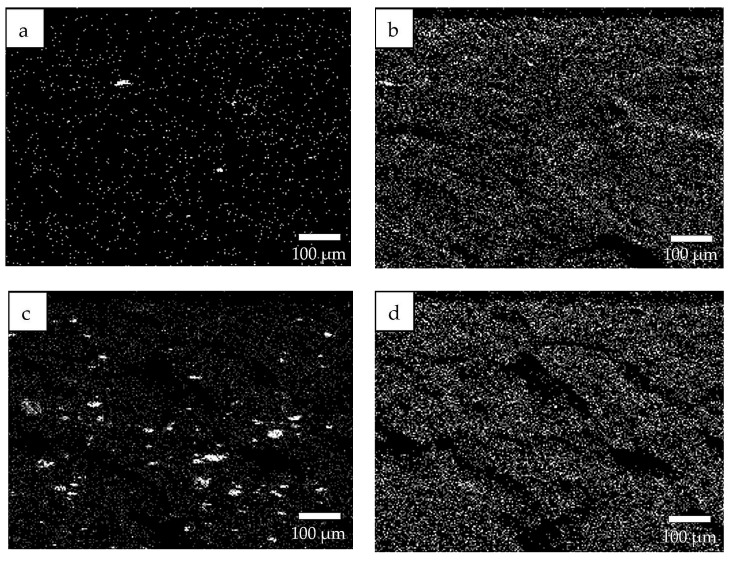
EDS data for the SKPO +UPTFE rubber composites (×50): (**a**) EDS of fluorine, 1 phr UPTFE, (**b**) EDS of oxygen, 1 phr UPTFE, (**c**) EDS of fluorine, 15 phr UPTFE, and (**d**) EDS of oxygen, 15 phr UPTFE.

**Figure 3:**
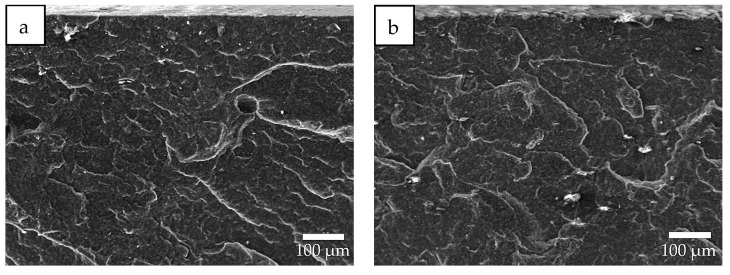
SEM images of SKPO + UPTFE rubber composites: (**a**) SEM, 1 phr UPTFE, and (**b**) SEM, 15 phr UPTFE.

EDS data for the SKPO-UPTFE rubber composite containing 1 phr UPTFE reveals a uniform distribution of UPTFE particles in the SKPO, which is shown in Figure 2a,b. The particle size of the local UPTFE aggregates is less than 10 µm, which is shown in Figure 2a. Because of the agglomeration, the particle size of UPTFE increases to 40 µm at 15 phr UPTFE, which is shown in Figure 2c. UPTFE particles are localized into agglomerates (>10 µm), which indicates weak interfacial interactions between SKPO and UPTFE. This is shown in Figure 2c,d. SEM images of the SKPO-UPTFE rubber composites are shown in Figure 3a,b, which can be seen to correspond to the EDS data shown in Figure 2.

**Figure 4:**
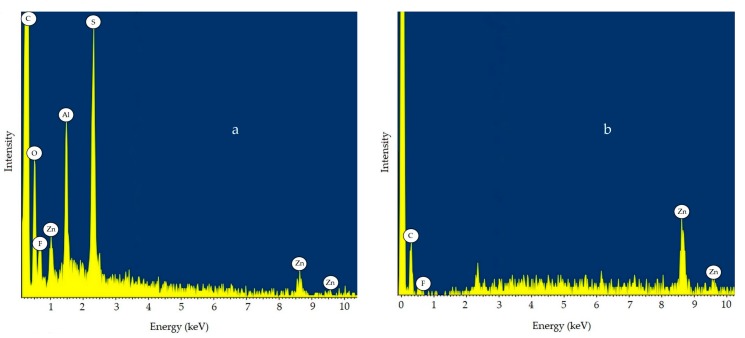
EDS spectrum for SKPO + 1 phr UPTFE rubber composite at: (**a**) surface of rubber composite and (**b**) interior of rubber composite.

The EDS spectra of an SKPO rubber composite containing 1 phr UPTFE reveals that the amount of fluorine on the surface of a rubber composite is greater than that inside the rubber composite, which is shown in Figure 4a,b.

This means that the UPTFE particles tend to concentrate on the surface of the rubber composite. The surface tension (σ) of PTFE is 19 mN/m while that of SKPO is 32 mN/m [26]. This data is consistent with the observations described above, which show that the PTFE and its lower surface tension, migrates to the surface of the rubber composite.

**Figure 5:**
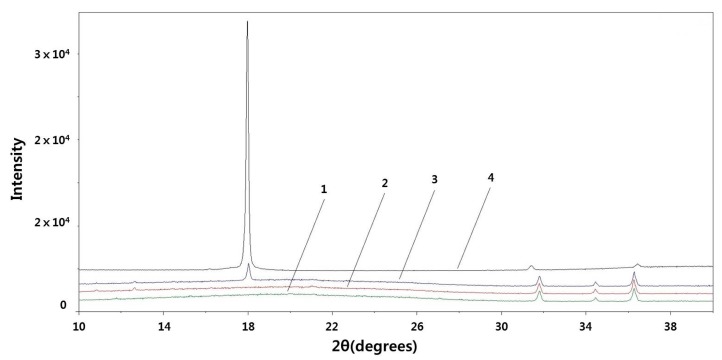
XRD patterns of: (1) initial SKPO rubber, (2) SKPO + 1 phr UPTFE rubber composite, (3) SKPO + 10 phr UPTFE rubber composite, and (4) UPTFE.

The surfaces of the SKPO and SKPO-UPTFE rubber composite were investigated by XRD analysis, which is shown in Figure 5. The SKPO containing 10 phr UPTFE exhibits a characteristic peak at a 2θ value of 18°, which originated from the UPTFE. On the other hand, there is no peak at 2θ = 18° in the case of the SKPO rubber composite containing 1 phr UPTFE. Therefore, to analyze the interior of the rubber composite containing SKPO and 1 phr UPTFE, another method such as X-ray photoelectron spectroscopy [27] would be required.

**Figure 6:**
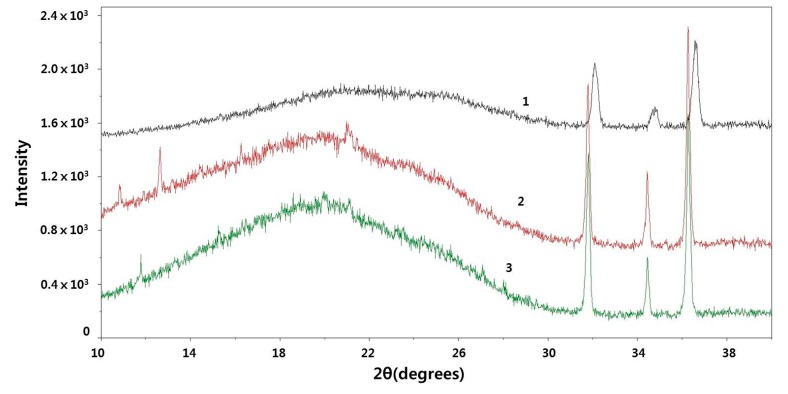
XRD pattern of SKPO + 1 phr UPTFE rubber composite: (1) interior of rubber composite, (2) surface, and (3) standard SKPO rubber.

The XRD patterns for the surface and interior of the SKPO rubber composite containing 1 phr UPTFE are shown in Figure 6. The XRD method fails to detect 1-phr UPTFE, which is shown in Figure 6.

New peaks at 2θ values of 13° and 11° are observed on the surface of this rubber composite. These peaks are assumed to originate from the low-molecular-weight fraction of the UPTFE during the vulcanization of the rubber because the low-molecular-weight fraction has a lower melting point and higher mobility and, therefore, can be mobilized at a processing temperature of 150 °C [12].

On the other hand, the three peaks at 2θ values of between 30° and 38° originate from ZnO (part of the curing system, 5 phr in the SKPO rubber). 

## 4. Conclusions

An SKPO rubber composite containing 1 phr UPTFE has the same mechanical and wear resistance properties as that addressed in our previous study, which contained 20 phr PTFE [9,10] but offers superior frost resistance. This significant reduction in the required amount of filler is a result of using smaller and, therefore, more uniformly distributed particles of UPTFE in the rubber matrix compared to that achieved with PTFE. Furthermore, weak interactions between the SKPO and UPTFE may be attributed to the difference in the physical and chemical properties such as the polarity, surface tension, and van der Waals forces between them. This leads to the UPTFE migrating towards the surfaces of the rubber composites. Since UPTFE has a low friction coefficient, the relatively high concentration of UPTFE on the surface minimizes the friction between the metal parts and can lead to the protection of the sealing materials during the operation of the machinery. Such rubbers would, therefore, be ideal for manufacturing rubber sealing materials intended for applications in extreme arctic conditions.

## 5. Patents

This rubber composite material has been patented in the Russian Federation and the United States [28,29].

## Figures and Tables

**Table 1:** Chemical Composition of SKPO rubbers (phr).

Ingredients	SKPO	SKPO + 20 PTFE	SKPO + UPTFE
SKPO	100	100	100
Stearic acid	1.0	1.0	1.0
Zinc oxide	5.0	3.0	3.0
2-mercapto-benzothiazole	-	2.0	2.0
Dibenzothiazyl disulfide	1.5	-	-
Thiuram disulfide	1.0	2.0	2.0
Phenyl-β naphthylamine	2.0	-	-
Carbon black P-803	60.0	60.0	60.0
Dibutoxyethyl adipate	10.0	-	-
Sulfur	1.5	1.5	1.5
PTFE	-	20	-
UPTFE	0	-	0.5, 1, 3, 5, 10

**Table 2:** Physical and Mechanical Properties of SKPO rubbers.

Properties	SKPO	SKPO + 20 PTFE	1	2	3	4	5	6
*f_P_* (MPa)	7.5	8.5	7.4	7.4	7.9	7.9	7.4	7.9
ε_p_ (%)	192	180	240	268	268	232	230	228
*f*_100_ (MPa)	4.9	5.8	3.3	3.2	3.2	3.2	3.6	3.7
*K_V_*	0.88	0.47	0.99	0.97	0.98	0.96	0.95	0.90
*C* (%)	58.3	53.5	54.0	52.0	52.0	62.0	63.0	68.0
Δ*V* (cm^3^)	0.17	0.11	0.11	0.10	0.12	0.13	0.14	0.15
Δ*Q (%)*	22.0	18.0	17.0	17.2	18.3	19.4	21.5	22.2

1: SKPO + 0.5 phr UPTFE, 2: SKPO + 1 phr UPTFE, 3: SKPO + 3.0 phr UPTFE, 4: SKPO + 5.0 phr UPTFE, 5: SKPO + 10.0 phr UPTFE, 6: SKPO + 15.0 phr UPTFE, *f_P_*: tensile strength, ε_p_: elongation at break, *f*_100_: tensile stress at 100% elongation, *K_V_*: frost resistance coefficient with tension at −50 °C, *C*: compression set at 100 °C and 72 h, Δ*V*: volumetric wear, Δ*Q*: degree of swelling at 70 °C and 72 h in oil.
